# Keeping the Centromere under Control: A Promising Role for DNA Methylation

**DOI:** 10.3390/cells8080912

**Published:** 2019-08-16

**Authors:** Andrea Scelfo, Daniele Fachinetti

**Affiliations:** Institut Curie, PSL Research University, CNRS, UMR144, 26 rue d’Ulm, 75005 Paris, France

**Keywords:** Centromere, epigenetic, DNA methylation, cell division, genome instability

## Abstract

In order to maintain cell and organism homeostasis, the genetic material has to be faithfully and equally inherited through cell divisions while preserving its integrity. Centromeres play an essential task in this process; they are special sites on chromosomes where kinetochores form on repetitive DNA sequences to enable accurate chromosome segregation. Recent evidence suggests that centromeric DNA sequences, and epigenetic regulation of centromeres, have important roles in centromere physiology. In particular, DNA methylation is abundant at the centromere, and aberrant DNA methylation, observed in certain tumors, has been correlated to aneuploidy and genomic instability. In this review, we evaluate past and current insights on the relationship between centromere function and the DNA methylation pattern of its underlying sequences.

## 1. Introduction

Cellular division is essential for proper organism development and growth. During cell division, the genome must be accurately segregated to each daughter cell. To accomplish this, eukaryotes attach chromosomes to the microtubule spindle and pull the sister chromatids toward the opposite spindle poles (reviewed in [1]). In this context, centromeres play a key role for proper chromosome inheritance; they are the chromosomal assembly site for the kinetochore, the protein complex mediating the spindle attachment and chromosome separation (reviewed in [2]). Defects in any of the pathways regulating centromere assembly give rise to chromosome mis-segregation. Chromosome mis-segregation, in turn, can promote structural alterations (such as chromosome rupture, rearrangements and translocations) and imbalance of chromosome numbers (an event known as aneuploidy), both hallmarks of cancer cells (reviewed in [3,4]).

The human centromere is a complex DNA/protein structure forming an atypical chromatin, whose epigenetic determinant is the histone H3 variant CENP-A (Centromere protein A). CENP-A is required for the assembly of the constitutive centromere associated network (CCAN), an association of centromeric components necessary to mediate the assembly of the kinetochore prior to mitosis (reviewed in [1]). CENP-A is present in all active centromeres independent of the underlying DNA sequence [1,5]. In particular, human centromeric chromatin assembles on α-satellite DNA sequences made of head-to-tail tandem repeats of AT-rich 171 bp-long monomers [6], overall accounting for approximately 3% of the genome. These monomers are additionally organized into higher-order repeats (HORs), spanning from 340 bp to 6 Kb [7]. Globally, HOR repetition builds large centromeric DNA domains of about 0.3–5 Mb. Centromeric sequences vary among chromosomes due to the variation in the number of repeated tandem monomers. However, individual monomers are characterized by 50–70% of sequence homology, while HORs can be up to 95% similar owing to their homogeneous α-satellite arrays [8,9]. A substantial portion of these repeats are occupied by Centromere protein B (CENP-B), which is the only known centromeric DNA sequence specific binding protein [10]. Other constituents of centromeric DNA are transposable elements, which have important roles on chromosome function and evolution (reviewed in [11]). The sequences flanking the centric locus of a centromere, called the pericentromere, are less organized and more heterogeneous. While pericentromeres acquire epigenetic features that are distinct from the centromeric ones (e.g., trimethyl histone H3 Lys9), they still maintain the repetitive nature, mostly given by divergent DNA monomers intermingled with retrotransposable elements such as LINE and SINE (long and short interspersed nuclear elements, respectively; reviewed in [11]).

The unique repetitive nature of the centromeric DNA sequence could confer to the centromere a complex DNA topology [12], making it a potentially fragile region of the genome. Indeed, several topological structures, such as DNA catenates and DNA loops have been proposed to accumulate at centromeric loci in physiological conditions. These structures require the intervention of specific DNA replication and recombination processes in order to preserve centromere integrity. Given this peculiarity, problems in any of these pathways can make the centromere a hot-spot for physical breakage events (reviewed in [3,13]). For this reason, centromere dysfunction is frequently associated to the formation of neoplastic lesions, as well as aging and specific human genetic disorders [3,13].

It is widely known that one of the most characteristic features of repetitive elements within the genome is their DNA methylation status. A prime example are transposable elements, which are repressed by DNA methylation to impede their mobility. Within the centromere, the α-satellite monomeric repeat contains three methylatable CpG di-nucleotides, two of which are present in the CENP-B box [10]. Given the repetitive nature of centromeres, it is tempting to hypothesize that DNA methylation might regulate centromere biology at different levels, thus contributing to genomic stability. In the following sections, we will discuss the current understanding of DNA methylation patterns at centromere, how they are established and maintained and how they may contribute to centromere function.

## 2. DNA Methylation and its Writers

DNA methylation is assumed to have a bi-functional role, i.e., regulation of gene expression and chromatin structure (reviewed in [14,15]). DNA methylation probably evolved as a way to preserve genomic integrity by defending the eukaryotic genomes from the random insertion of parasitic elements [16]. In mammals, DNA methylation is a heritable epigenetic mark essential for organism development and homeostasis, and it is typically associated with transcriptionally inactive regions of the genome. The primary methyl modification of DNA is 5-methylcytosine (5mC) resulting from the covalent attachment of a methyl moiety to the C-5 position of cytosine in the context of CpG dinucleotides, which are often present as repetitive dinucleotides (reviewed in [14]). The reaction is catalyzed by the activity of methyltransferase enzymes DNMT1, DNMT3A and DNMT3B, which cooperate to maintain global DNA methylation patterns. In addition, other non-canonical DNMTs exist, i.e., DNMT2 and DNMT3L [17,18]. Although these proteins are homologous to DNMT3A and -3B, they are devoid of any enzymatic activity.

De novo deposition methyl moieties are mediated by DNMT3A and DNMT3B, which target newly integrated host sequences and both unmethylated and hemimethylated CpG substrates. These methyltransferases are required for DNA methylation during early development, especially after the global demethylation of the genome in the pre-implantation embryo [19,20]. Moreover, DNMT3C has been recently identified as a de novo methyltransferase acting on promoters of evolutionarily young retrotransposons in the male germline [21]. DNMT1 is able to maintain pre-established DNA methylation patterns over cell divisions and it has high affinity for hemimethylated DNA sequences; however, DNMT3A and -3B were also proposed to be necessary to preserve already established methylation patterns (reviewed in [22,23]). A role for DNMT1 in de novo methylation cannot be excluded, especially in the context of repetitive elements. Indeed, the analysis of DNA methylation patterns by high-resolution sequencing of repetitive elements (including major satellites) unveiled a de novo methylation activity of DNMT1 at those regions [24]. A putative role of DNMT1 in the establishment of methylation at specific subtelomeric repeat of mouse chromosome 4 has also been reported [25]. Moreover, in vitro studies in murine model have demonstrated that DNMT1 is able to deposit methylation moieties on unmodified DNA when in the presence of DNMT3A [26], and to establish de novo CpGi hypermethylation on the tumor-associated genes promoter in human cancer cells [27]. DNMT3L may also regulate DNA methylation at centromere. Although it has no enzymatic activity, it acts as cofactor for DNMT3 [28,29]. DNMT3L bears a cysteine-rich ADD (ATRX–DNMT3–DNMT3L) domain, which is able to bind to histone H3 unmethylated on lysine 4 [30,31,32]. Because this modification is not present at pericentromeres, DNMT3L may bind and mediate the recruitment of canonical DNMT3 allowing the establishment of methylation pathways at these loci.

Patterns of 5mC are specifically set during embryogenesis and are re-shaped during lineage commitment and differentiation processes, ultimately leading to specific patterns in somatic cells (reviewed in [33]). Indeed, alterations of imprinted configurations of DNA methylation during development give rise to human genetic diseases like Prader–Willy syndrome (reviewed in [34]).

Most of mammalian CpG dinucleotides (around 70–80%), which are abundant in satellite DNA, repetitive elements and non-repetitive intergenic DNA (reviewed in [35]), are methylated and transcriptionally repressed [36]. CpG dinucleotides are further present in about half of the mammalian gene promoters, where they are mostly unmethylated and constitute the so called CpG islands (CGIs). CGIs can undergo DNA methylation during development, leading to transcriptional silencing of the corresponding promoter thus granting tissue-specific expression [37]. At gene bodies, CpG methylation is less frequent and this correlates with gene activation [38]; gene body methylation outside CGIs occurs to silence transposons [16], and to control alternative splicing [39].

Beyond controlling gene transcription, DNA methylation has been shown to model chromatin structure, consequently influencing the accessibility of DNA damage machinery and the recruitment of DNA methyl-binding proteins (reviewed in [33]). In pathological conditions, hypomethylation at satellite and repetitive elements of the genome and the hypermethylation of CGIs within promoters of onco-suppressors genes are causative of genome instability that favors the establishment of neoplastic lesions (reviewed in [22]).

CpG within centromeric DNA sequences are strongly enriched in DNA methylation, which is tightly maintained throughout cell cycle progression [40]. Although the presence of high levels of DNA methylation at the (peri)centromere is widely proven, whether the different DNMT enzymes act specifically or redundantly in the deposition of this epigenetic mark remains controversial. Dnmt3a and Dnmt3b knock-out mice bear different developmental defects, suggesting different patterns of DNA methylation throughout the genome, probably involving also the centromeric sequences; indeed, minor satellites in Dnmt3b-/- mice showed an increased methylation loss, while those of Dnmt3a-/- mice were poorly or not at all affected [19]. This could suggest that DNMT3A and -3B may have different specificities for major or minor satellites. This hypothesis is in agreement with the preferential association of DNMT3A (rather than DNMT3B) to pericentromeric regions [41]. While the role of DNMT1 in maintaining global methylation is widely known, whether it is involved in the maintenance of (peri)centromere methylation is an open question. Since DNMT1 has been shown to continuously associate with heterochromatin in a replication-independent manner, this would also allow the maintenance of the packed methylated status of the centromeric heterochromatin [42]. It is also plausible that DNMT3A and DNMT3B may contribute to maintaining DNA methylation at the centromere, as both enzymes were shown to be involved in global methylation maintenance at least in mouse embryonic stem cells (mESCs) [43].

The CpG methylation landscape has been investigated employing methyl-sensitive restriction enzymes, fluorescence-based techniques and DNA sequencing (mainly performed after bisulfite conversion). However, assessing DNA methylation at the centromere through next generation sequencing approaches is quite challenging due to its high-repetitive nature and the incomplete annotation of centromeric sequences.

## 3. Mechanisms Maintaining DNA Methylation at (Peri)Centromeric Regions

**Figure 1:**
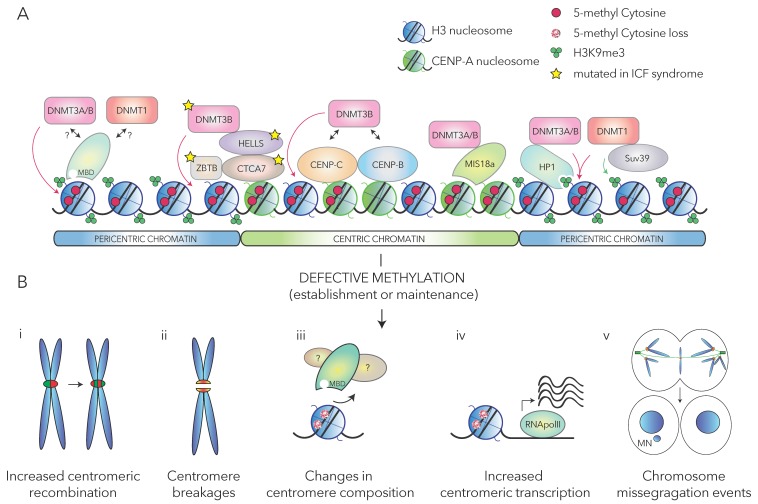
(**A**) Schematic representation of some proposed mechanisms of DNA methylation establishment and maintenance at (peri)centromeric regions. Centromeric proteins CENP-A, -B, -C recruit, by direct or indirect interactions, the indicated DNA methyltransferase enzymes to centromeric chromatin. At pericentromeric loci, DNMT1 is recruited by Suv39 proteins exerting the deposition of tri-methylated H3Lys9: the latter, in turn, is bound by HP1 proteins mediating the recruitment of DNMT3A/-3B. 5-methyl Cytosine is recognized by methyl-binding domain-containing proteins, which can possibly recruit DNMTs enzymes. The proteins involved in ICF (Immunodeficiency, Centromere instability, Facial anomalies syndrome) etiology are also shown. With the exception of DNMT3B, whose mutation does not affect α-satellite methylation, ICF-specific mutations within the indicated proteins (namely, ZBTB, CTCA7, HELLS) cause hypomethylation at both pericentromere and centromere regions. (**B**) Proposed consequences of defective methylation at (peri)centromere. Loss of centromeric methylation may lead to: (i) increased rate of recombination of centromeric repeats; (ii) increased centromere breakages; (iii) displacement of methyl-binding proteins and their interactors, thus inducing changes in the overall proper centromeric protein network; (iv) increased generation of α-satellite transcripts leading to genomic instability; (v) chromosome mis-segregation events due to defective centromere/kinetochore assembly and/or premature cohesion loss, with the eventual generation of micronuclei (MN).

Given the peculiarity of centromeres, it is expected that DNA methylation maintenance mechanisms differ from those acting on the rest of the genome. Figure 1a illustrates the main proposed factors responsible for DNA methylation maintenance at (peri)centromere. However, little is known about how the different centromeric components contribute to establish and maintain DNA methylation at centromeric regions.

A link between centromeric proteins, such as CENP-B, and the methylation of underlying DNA sequences has been observed in several studies. CENP-B is the only centromeric protein bearing sequence specific DNA binding properties, specific for a 17 bp sequence, namely CENP-B box, present in about 30% of all human α-satellites [10,44]. Interestingly, the CENP-B box contains two of the three CpG di-nucleotides present in the whole α-satellite consensus sequence [10]. In vitro studies show that CENP-B association to CENP-B box is methylation-sensitive due to a steric clash between DNA methyl moieties and side chains of Thr44 and Arg125 of CENP-B [45]. Accordingly, anomalous DNA methylation upon 5-azacytidine (AC) treatment caused erroneous CENP-B binding or even its displacement [46]. A study of centromeric assembly on human artificial chromosome (HAC) carrying alphoid DNA showed that, once chromosomally integrated, CENP-B enhances CpG methylation and H3K9me3 deposition on the HAC to prevent CENP-A incorporation; however, the methylation status of the native CENP-B binding sites remained unaltered [47]. This points towards a role for CENP-B in the establishment of precise patterns of DNA methylation of centromeric sequences, which in turn are likely necessary to assemble proper chromatin composition at the (peri)centromere. Whether defined DNA methylation patterns at (peri)centromeric regions are also fundamental to assemble centromeric proteins and their interacting partners needs further investigation.

Yeast two-hybrid screening and immuno-precipitation assays revealed an association between DNMT3B and the centromeric protein CENP-C [48]. However, CENP-C knock-down induced only a ~20% DNA methylation reduction at α-satellite and pericentromeric satellite 2. This study also showed an altered DNMT3B localization and increased transcription at centromeric loci upon CENP-C reduction. Chromosome segregation errors were observed in colorectal cancer cells following both CENP-C and DNMT3B reduction; these mitotic defects have been proposed to be a consequence of increased production of centromeric transcripts [48]. Nevertheless, further studies are required to assess a functional link between CENP-C and DNMT3B in the overall maintenance of centromeric methylation.

Factors involved in the centromeric chromatin assembly may further contribute to DNA methylation maintenance at underlying sequence. Mis18α, known to mediate the centromeric recruitment of de-novo synthesized CENP-A [49] was shown to directly interact with DNMT3A and DNMT3B in mouse cells [50]; loss of Mis18α led to modest decrease in minor-satellite methylation (assessed by bi-sulphite sequencing and methylation-sensitive DNA digestion), suggesting that Mis18α may help tether DNMT3A and DNMT3B to the centromere thus maintaining the methylation levels. The authors further claimed the compromised centromeric localization of CENP-A (observed in around 60% of cells depleted for DNMT3A and -3B) to be a consequence of DNA methylation reduction at minor-satellite [50]. However, it cannot be ruled out whether CENP-A mis-localization after DNMT3s knockdown was a direct consequence of either altered DNA methylation landscape or anomalies in Mis18α function and/or localization, as the authors show that DNMT3s/ Mis18α localization is mutually dependent.

The nucleosome modification landscape of pericentromeric chromatin also have an effect on the DNA methylation process. Evidence comes from the altered methylation of major satellites observed in mESCs upon deletion of Suv39h1 and Suv39h2, the enzymes responsible for H3K9me2/3 deposition [51]. In this background, and in line with a previous report showing Suv39h and DNMT3B interaction [52], Suv39 proteins were crucial to recruit DNMT1 and UHRF1, which cooperatively maintain DNA methylation at major satellites [51,52]. In contrast, recruitment of de-novo methyltransferase DNMT3A and -3B was unaltered [51]. In the same study, DNA methylation was shown to favor tethering of major satellites to the nuclear lamina, as demonstrated by chromatin reorganization upon simultaneous depletion of the three DNMT enzymes [51].

Association of pericentromeric chromatin to the nuclear periphery can have a fundamental role in the maintenance of genomic stability. The interdependence between H3K9me3 and DNA methylation at (peri)centromere can be further reinforced by other mechanisms, for example the NoRC (nucleolar remodelling complex) activity. Indeed, the NoRC component TIP5 stimulates H3K9me3 deposition at major and minor satellite repeats and interacts with CENP-A [53]. Another protein family probably involved in the maintenance of DNA methylation at the (peri)centromere is the heterochromatin protein 1 (HP1), which is composed in mammals by three protein isoforms, namely HP1α, HP1β and HP1γ. Through the binding of methylated H3K9, mediated by their chromodomain [54], they localize to the (peri)centromere, where HP1α and HP1β were shown to interact with DNMT3A and -3B [52,55]. They exert a structural function, as they constrain the centromere in heterochromatic foci called chromocenters during the interphase [54]. Although the (peri)centromere DNA hypomethylation did not affect HP1 localization, as observed in ICF (Immunodeficiency with Centromere instability and Facial anomalies syndrome) patient-derived cells [56], whether HP1 at the (peri)centromere can stimulate DNA methylation is poorly understood.

It is plausible that other, so far unidentified, proteins could contribute to the maintenance of centromere features in a DNA methylation-dependent manner. Indeed, like in the case of CENP-B, we can envisage the presence of proteins whose binding to (peri)centromere loci (including transcription factors) occurs in a DNA methylation-dependent manner, thus leading to the specification of precise protein assembly required for centromere function.

Additional evidence of the link between centromere chromatin composition and DNA methylation is given by the ATRX protein, member of the SNF2 family of helicase/ATPases, which is required for several cellular processes including H3.3 deposition at the (peri)centromere when in complex with DAXX [57]. In patients affected by ATRX syndrome (X-linked α-thalassaemia mental retardation), a set of satellite repeats at centromeric and sub-telomeric regions were shown to be aberrantly methylated [58]. Variations in DNA methylation could indirectly impair correct ATRX localization through the interaction with MECP2, which contains a methyl-CpG-binding domain (MBD). The MBD domain, indeed, mediates both MECP2 localization to the (peri)centromere [59] and its interaction with ATRX [60]. Although ATRX contains a plant homeodomain-like zinc finger domain, shared with de novo methyltransferases DNMT3A and -3B, its direct role in (peri)centromeric DNA methylation needs further study.

## 4. DNA Methylation and the Maintenance of Centromere Features and Stability

(Peri)centromeric chromatin is densely packed and needs to be robust enough to absorb the mechanical stress caused by the pulling of the spindle microtubules on the kinetochore. Several lines of evidence point towards the existence of an effect of DNA methylation on (peri)centromeric chromatin structure and function. However, whether a direct functional link between centromere dysfunction and centromere hypomethylation exists is still a matter of debate and investigation (Figure 1b illustrates the main putative roles of DNA methylation on the maintenance of centromere functions).

### 4.1. The Impact of (Peri)Centromeric DNA Methylation on Genomic Stability

The hypothesis that genomic stability is also maintained by DNA methylation is supported by loss of function studies of DNMT1 or DNMT3A/-3B activity. In addition to triggering decreased levels of centromeric DNA methylation [19,61,62], the loss of DNMTs enzymatic activity leads to increased genomic instability [63,64]. However, it was never demonstrated if the resulting genomic instability was a direct effect of methylation loss at (peri)centromere, global genomic hypomethylation or if hypomethylation of centromeric sequences results in a dysfunctional centromere. DNMT3B—but not DNMT3A—depleted mouse embryonic fibroblasts (MEFs) exhibited modest chromosomal abnormalities, characterized by the presence of fused broken chromosome ends and anaphase bridges [64]. In contrast, triple Dnmt1/-3a/-3b knock out mESCs are viable, with unaltered pericentromeric epigenetic features and no apparent defects in chromosome segregation and condensation [65]. This may add an additional layer of complexity, because DNA methylation could function differently in the overall maintenance of genomic stability in pluripotent and differentiated cells. Moreover, different patterns and levels of centromeric methylation may exist in pluripotent and lineage-specific differentiated cells.

Insight into the relationship between centromere dysfunction, DNA hypomethylation, and genomic instability come from observations on the genesis of micronuclei. Micronuclei (MN), typical of cancer cells, normally arise from chromosome segregation errors, and are associated with genomic instability. The DNA entrapped in MN is known to be more prone to breaks and rearrangements due to a defective nuclear envelope [66,67,68]. It has been proposed that chromosome segregation into MN can be a consequence of hypomethylation of (peri)centromeric regions [69,70]. Indeed, loss of methylation at satellite 2 and 3 has been associated to DNA elongation, even if the underlying mechanisms were not addressed [69]. One possibility is that it weakens the kinetochore tension, thus affecting proper microtubule attachment and chromosome segregation, with consequent generation of MN [71]. Accordingly, cell treatment with the DNA demethylating agent AC caused the preferential inclusion into MN of specific human chromosomes, namely 1, 9, 15, 16, and Y [70].

Among the effects of DNA methylation on the maintenance of genome stability is its proposed ability to control crossover and recombination at the centromere during meiosis and mitosis. Centromeres are known “cold spots” for recombination. Repression of crossovers at centromeric regions, which occurs through different mechanisms, is known as the “centromere effect” (reviewed in [72]). When crossovers occur at juxtacentromeric regions, defects in chromatid segregation during the second meiotic event have been observed in different species, including Drosophila [73], humans [74], plants [75] and yeast [76]. Thus, in physiological conditions, crossovers must be inhibited at centromeres to avoid chromosome breakage and loss following impaired cohesion of sister chromatids, which cause their premature separation and mis-segregation (reviewed in [77]). In this context, DNA methylation may serve as a protective factor, but the assessment of crossover frequency in eukaryotes is quite challenging principally owing to the difficulty of following many meiotic events. However, studies in the plant Arabidopsis thaliana have shown that DNA methylation loss favors crossovers at the centromere, while inhibiting the ones occurring at euchromatic chromosome arms [78,79,80]. This may be due to compensatory effects rising right after DNA hypomethylation establishment and/or to changes in the surrounding epigenetic patterns. Whether this mechanism is conserved in eukaryotes needs further investigation. These observations are further corroborated by the increased recombination specifically scored at centromeric regions upon defective DNA methylation during mitosis [81]. Using centromeric chromosome-orientation fluorescent in situ hybridization (Cen-CO-FISH), Jaco and colleagues described centromeres as recombinogenic sites when compared to other genomic loci in murine cells in presence of low level of methylation [81]. The frequency of centromere recombination was enhanced upon depletion of either DNMT1 or DNMT3A/-3B; moreover, DNA methylation loss was accompanied by a reduction in the length of minor satellite repeats, measured by quantitative FISH (Q-FISH) [81].

DNA methylation may also play a role in centromere evolution. Centromeric repeats have the highest evolving rate within the eukaryotic genome. Nevertheless, as described above, they are a “cold spot” for chromosome homologous recombination during meiosis, which normally allows to shuffle genomic loci between chromosome pairs. It has been proposed that centromeres evolve thanks to particular mechanisms, such as gene conversion and unequal exchange in satellite arrays (reviewed in [77]). It can be speculated that centromeric DNA methylation may also control the mechanisms underlying centromere diversification, similarly to what is proposed for crossovers and recombination. Studies in fish have unveiled that centromeres bearing different levels of methylation are subject to a different evolutionary pressure [82].

### 4.2. The Role of DNA Methylation in the Regulation of (Peri)Centromeric Features

DNA methylation may also have an impact on chromosome condensation and stiffness, mainly mediated by condensin I and II complexes [83,84]. Indeed, depletion of the condensin complex induced chromatin decondensation [85], similar to what was observed upon AC-induced DNA demethylation [86]. Although removal of condensin I and II complexes did not seem to induce pericentromeric hypomethylation in mESCs [87], an interaction between DNMT3B and condensin complexes has been found [88]. This suggests that any of the two condensin complexes can promote DNA methylation (maintenance or de novo deposition) and stability of (peri)centromeric regions through DNMT3B recruitment. Moreover, hypo- or demethylation of pericentromeric regions seem to correlate with lack of centromeric cohesion; it has been proposed that DNA methylation may interfere with chromatid cohesion, regulating the interactions between condensin, cohesin and DNA [89].

DNA methylation can alter the structure and accessibility of chromatin. DNA methylation is able to induce a closed chromatin conformation in a sequence-independent manner, with consequences for nucleosome stability and dynamics (such as changes in nucleosome positioning and assembly) [90,91,92]. Nevertheless, the precise mechanisms of action are still matter of investigation. Moreover, human lymphocytes treated with the DNA hypomethylating drug AC showed severe defects in chromosome condensation. The hypomethylation induced uncoiling of satellite DNA containing chromatin (pericentromeric chromatin) led to breakpoints and consequent rearranged chromosomes appearing in the next cell generation [86]. However, the condensation defects were demonstrated to likely be dependent on the satellite types, with the α-satellite being less affected by hypomethylation [93]. DNA methylation loss may alter the steric configuration of the chromatin, consequently modifying the array of proteins that mediate condensation. DNA methylation may have an additional role in the maintenance of structural features of nucleosomes at (peri)centromere. Crystal structures of nucleosomes assembled on type 2 and type 3 satellites revealed that methylated sequences favored the formation of regular folded heterochromatin, which, in vivo, can contribute to maintain genome stability [94]. Overall, whether and how DNA methylation could influence the structural properties of centromeres is still an open question.

In order to maintain proper centromeric condensation and structure, it is conceivable that DNA methylation also cooperates with specific configurations of histone post translational modifications (PTMs), which are known to be different at centromeric and pericentromeric chromatin. Indeed, cross-talk mechanisms are widely proven to establish precise PTM patterns throughout the genome. Pericentromeric chromatin mainly resembles a classic heterochromatin landscape, abundant in H3K9me2/3, H4K20me3, H3S10ph, and characterized by low levels of histone acetylation. On the other hand, centromeric chromatin, although densely packed, is devoid of H3K9me3 but is enriched in H3K4me2, which is a typical PTM permissive for gene transcription [reviewed in [95]. The function at centromere of this chromatin mark is proposed to facilitate the chaperon HJURP in CENP-A assembly as demonstrated by assembly assays on a synthetic human kinetochore [96]. Notably, this is in agreement with the transcriptional activity of certain centromere regions, which is likely required for proper centromere functionality (reviewed in [97]). How, and if DNA methylation cross-talks with chromatin PTMs is unclear. The importance of a proper cooperation between DNA methylation and histone PTMs patterns is highlighted by the severe defects in chromosome alignment and segregation observed upon depletion of the histone methyltransferases responsible for the deposition of the major (peri)centromeric PTMs. In particular, it has been demonstrated that pericentromeric histone methylation, occurring during G2 phase, is critical to provide the structural integrity of pericentric heterochromatin prior to mitosis [98]. Similarly, inhibition of Histone DeACetylase (HDAC) activity leads to chromosome condensation and segregation defects [99]. Interestingly, the maintenance of pericentromeric hypoacetylation also depends on DNMT1. A molecular interaction between HDAC and DNMT1-DMAP (DNMT1-Associated Protein) complexes was reported. Although the two complexes were independently targeted to the (peri)centromere, they were both required for maintaining H3K9me3 levels and HP1 binding. This event highlights a cooperative cross-talk between the two pathways in the preservation of pericentromeric features. [100]. Therefore, impaired DNMT1 activity may also lead to genomic instability due to defects in maintaining proper pericentromeric PTMs.

A study performed in human embryonal carcinoma cells reported that DNA methylation also has an effect on the deposition of pericentromeric mark H3S10P catalyzed by Aurora-B kinase during G2 phase, whose function is to guarantee controlled chromosome condensation, segregation and cytokinesis [101,102,103]. The authors observed that inhibition of DNA methylation impaired Aurora-B recruitment and formation of H3S10P foci at (peri)centromere, with a consequent reduction of the number of mitotic cells [101]. However, it is unlikely that Aurora-B is recruited at the (peri)centromere by direct recognition of DNA methylation, rather, DNA methylation may render the chromatin context permissive for its recruitment. Given the dynamics of Aurora-B binding at the (peri)centromere, in addition to DNA methylation, other factors should exist in order to regulate this mechanism.

AC-induced DNA demethylation of (peri)centromeric chromatin in differentiated cells caused H3.3 deposition and accumulation of euchromatic features, accompanied with transcriptional activation of centromeric satellite repeats [104]. Uncontrolled accumulation of small murine minor satellite transcripts has also been observed to cause mis-localization of kinetochore complex, defects in sister chromatids cohesion and proper chromosome segregation [105]. Regression studies performed in human breast cancer specimens highlighted a correlation between overexpression of α-satellite transcripts, DNA hypomethylation and increased chromosome instability [106]. On the whole, centromeric transcription can be thought of as an additional way to maintain centromere functions, and DNA methylation may exert a fundamental role in tuning the generation of α-satellite transcripts.

### 4.3. DNA Methylation in the Maintaince and Establishiment of Centromeres

The possible impact of DNA methylation on centromere functions can be deduced from the neocentromere formation process. Neocentromere formation is a rare event observed in many species, including humans, which takes place on atypical chromosome sites, often nearby the original location, and gives rise to functional centromeres having similar properties to the conventional ones (reviewed in [107]). Neocentromeres can form in a sequence-independent manner, in absence of α-satellite repeats (reviewed in [108]). Whether a specific pattern of DNA methylation is required for a neocentromere to form is under debate. The acquisition of methylation on DNA sequences underlying a neocentromere may either be an epigenetic pre-requisite or a consequence of neocentromere formation, but these two options are hard issue to discern. Similarly, it would be interesting to determine if any changes in DNA methylation levels occur after centromere inactivation events. Newly acquired centromeres displayed increased DNA methylation [40]. Moreover, AC treatment of neocentromere-carrying cells displayed a modest increase of anaphase defects and bridging chromosomes involving the neocentromere, without affecting CENP-A levels [40]. These findings support the idea that DNA methylation may be necessary to favor the maintenance of a functional neocentromere and surrounding chromatin landscape. However, the drug treatment employed to induce DNA demethylation is known to have secondary effects, and it may alter the expression of proteins involved in centromere function, thus leading to the observed phenomena. 

Whether DNA methylation is essential for centromeric functions could be also inferred from analyzing the early development processes. Embryo reprogramming after fertilization implies a broad and dramatic epigenetic “resetting”. Of note, in the post fertilization phase, and during pre-implantation development, the paternal genome undergoes massive active DNA demethylation except at (peri)centromeric regions in human and many other species. On the contrary, the maternal genome retains DNA methylation along the entire chromosome arms (reviewed in [109]). As such, maternal chromosome methylation may account for an important function of DNA methylation at centromere. Retaining centromere DNA methylation at this stage may be crucial to maintaining and/or establishing proper centromere features. On the contrary, germ-line cells are characterized by global hypomethylation, even at (peri)centromere. Such low methylation levels have been suggested to be a favorable background for meiosis, the germ-cell specific chromosome segregation, supporting the assemblage of meiosis-specific kinetochore proteins [110].

## 5. DNA Methylation and Centromere Stability: Insights from Pathologies

Several types of pathologies provide evidence on the importance of (peri)centromeric DNA methylation in maintaining genomic stability. The most crucial examples are some tumor types and the ICF syndrome, the only known monogenetic disorder involving constitutive alteration of DNA satellite methylation patterns [62,111,112].

Among the common features of cancers, their altered karyotype is often associated to bulk hypomethylation of the genome, frequently accompanied by hypermethylation of tumor suppressor family gene promoters. Hypomethylation preferentially occurs at repetitive and (peri)centromeric regions, which are targets of recombination and breakage, found in 40–60% of tumoral cell lines by extensive SKY/CGH analysis [3,13]. In this context, as previously discussed, DNA methylation has been proposed to have a direct role in the suppression of centromeric rearrangements, by acting as an inhibitor of recombination [81]. The length of centromeric satellite repeats is critical for the recruitment of a proper network of centromeric proteins, thus ensuring centromere functions (our unpublished data and [113,114,115,116]). Further evidence was found in hepatocellular carcinomas showing that chromosome 1 rearrangements at pericentromeric levels correlated with hypomethylation of the corresponding type2 satellite repeats [117]; similarly, in a correlative study performed on ovarian epithelial carcinomas, breast adenocarcinomas and Wilms tumors, a strong association between hypomethylation of satellite 2 repeats and pericentromeric rearrangements was shown [118]. The same sequence-specific hypomethylation at juxtacentromeric regions was at chromosomes 1, 9, and 16 and the distal part of the long arm of the Y chromosome [119].

Many kinds of cancers display hypomethylation of satellite type 2 and 3, predominantly found at the (peri)centromeres of chromosome 1, 9 and 16 [120], a feature found also in cells from ICF syndrome patients. DNA methylation-dependent genomic instability in cancer cells can also be also a consequence of mutations affecting DNMT3B protein within its domain mediating its association with CENP-C (see section “Mechanisms maintaining DNA methylation at (peri)centromere regions”).

ICF syndrome is a rare autosomal recessive disorder whose clinical signs include primary immunodeficiency, mild facial anomalies and developmental delay accompanied by intellectual disability [121]. From a cytogenetic perspective, the disease is characterized by centromere instability causing multiradial chromosomal structures, chromosome decondensation and rearrangements occurring at (peri)centromeric regions [62]. The latter have been scored in the karyotype-based diagnosis performed on mitogen stimulated lymphocytes isolated from patients [121], found in combination with alterations in the nuclear organization [122,123]. The observed chromosomal instability is the consequence of DNA methylation loss on pericentromeric type 2 and 3 satellite repeats., although in further studies, the centromeric chromatin was also found to be hypomethylated [120,124]. DNA methylation loss is caused by mutation in the DNMT3B gene, which is present in homozygosity or heterozygosity in about half of the patients (classified as genotype ICF 1) [62].. It is worth noting that chromosomal rearrangements of ICF patients are similar to those observed in normal cells upon AC-hypomethylating treatment [125,126,127], suggesting a link between loss of methylation and chromosomal aberrations. From a molecular perspective, the mutation within DNMT3B gene occurs at the level of the PWWP domain, disrupting its capacity to target the enzyme to pericentric heterochromatin [41,128]. However, the DNMT3B PWWP domain binds to a 234-bp element of the pericentric chromatin and to random genomic sequences with the same efficiency, suggesting that it acts as nonspecific DNA-binding module [41].

Mutation in DNMT3B covered only 60% of the cases of ICF syndrome. More recent studies, taking advantage of new methodologies based on whole-exome sequencing, were able to identify mutations in other genes, thus explaining the lack of DNMT3B mutation in the rest of the ICF patients. These mutations affect the transcription factors ZBTB24 (genotype ICF 2) [129], CDCA7 (ICF 3) and the chromatin remodeler of the SWI/SNF family HELLS (Helicase Lymphoid Specific, alias LSH) (ICF 4) [130]; all these proteins are ubiquitously expressed in adult tissues. Patients with unidentified gene mutations were also reported and ascribed as ICFX genotype [124]. The four different genotypes were shown to bear diverse DNA methylation patterns, with ICF 2, 3 and 4 having aberrant methylation on heterochromatic CpG-poor genes involved in neuronal development, consistently with the clinical signs of the patients [131]. The only unifying exception was the hypomethylation of pericentric satellite-2 and-3 repeats and inactivated X chromosome in female patients [131]. Contrarily to ICF 1, ICF 2, 3 and 4 patients show major hypomethylation at α-satellite [129,132]. This strongly suggests the non-random nature of DNA methylation profiles at (peri)centromeric regions, established through specific regulatory mechanisms of DNMT activities, which are still poorly understood.

Although HELLS was already known to contribute to DNA methylation at murine centromeric repeats through direct interaction with DNMT3B [133,134], a role for ZBTB24 and CDCA7 in DNA methylation maintenance at murine centromeric repeats was only recently elucidated [130]. HELLS and CDCA7 act as a complex, namely CHIRRC (CDCA7-HELLS ICF-Related nucleosome Remodeling Complex), to regulate nucleosome structure, likely modulating its accessibility to DNA methyltransferase activities [135]. HELLS also contributes to the maintenance of a closed conformation of repetitive elements preventing the recruitment histone acetyltransferases [136,137]. In addition, a role for ZBTB24 in the positive transcriptional regulation of CDCA7 has been demonstrated both in mouse and human [131,138,139]. Methylation reduction of the pericentromeric satellite 1 scored in a Zebrafish model of ICF 2 (ZBTB24 mutation) elicited an increased generation of satellite transcripts, which in turn activated innate immunity [134]. It will be interesting to address whether this phenomenon contributes to the clinical signs of ICF patients, or to the etiology of some autoimmune pathologies.

A typical feature of ICF-derived cells is genomic instability, suggesting that defects in DNA repair pathways are also present. Indeed, HELLS protein is involved in the efficient resolution of double strand breaks (DSBs) [140], and a recent study has unveiled a direct connection between CDCA7 and non-homologous end joining (NHEJ) proteins Ku70 and Ku80 [141]. Mutation of CDCA7 (R274C, ICF3) disrupts the interaction with these NHEJ factors triggering chromosome mis-segregation and accumulation of γH2A.X, revealing the presence of persistent DNA damage [141]. Another factor likely contributing to the genomic instability in ICF cells is the presence of RNA:DNA hybrids, otherwise known as R loops, which have a deleterious effect on the maintenance of genome integrity. Indeed, an increased occurrence of R loops was observed at telomeres of ICF1 cells, and this has been correlated with chromosome end DNA damage [134]. Given that R loops accumulate at the centromere [142,143], it is likely that ICF mutations induce R loops accumulation and satellite repeats instability.

On the whole, further studies are required to address the exact mechanisms through which ICF factors, alone and/or in cooperation, maintain chromosomal stability. It is conceivable that these mechanisms involve other molecular machineries, as suggested by the link with DNA repair pathways. In particular, it is still to be tested if the recently identified ICF proteins directly contribute to the establishment of DNA methylation patterns, and whether they act specifically or redundantly in targeting their genomic loci. Similarly, the mechanisms how ICF factors mutations could lead to centromere dysfunction or alteration in its overall structure remain to be unveiled.

## 6. Concluding Remarks

The idea of a functional link between DNA methylation and centromere identity, function and integrity is very appealing. The importance of this relationship is strengthened by the genetic etiology of certain rare diseases, which establish a causal link between DNA methylation and centromere biology. DNA methylation may favor the establishment of a correct architecture and topology of the centromere, potentially being one of the epigenetic factors that contribute to control the binding of the centromeric protein CENP-B and CENP-C. Overall, this could explain the high level of chromosomal instability observed in cancer cells following DNA hypomethylation. Genomic instability of tumoral cells may derive from increased chromosome missegregation and aneuploidy due to mis-regulation of centromere function.

A particular aspect of centromeres is the presence of DNA damage factors [51,144]. It would be interesting to understand if DNA methylation also plays role in the modulation of DNA damage response at centromeres. Indeed, DNMT1 protein is rapidly but transiently recruited at damage sites upon double strand break (DSB) formation, probably acting as a sensor and maintaining DNA methylation levels at repaired loci [145]. Thus, DNA methylation at centromere could also favor the maintenance of genomic stability in the context of DNA damage; however, heterochromatic sites are believed to be a barrier for the accessibility of repair machineries.

The study of DNA methylation function specifically at centromere is challenging. Hypomethylation-inducing events, mainly relying on DNMTs depletion or demethylating drugs known to carry many side effects, act globally but not specifically at centromere, thus making it hard to discern non-specific effects. Much progress has been made during the last years to develop techniques able to profile DNA methylation patterns throughout the genome with high fidelity and resolution; in spite of this, when referring to centromere, the lack of complete human centromere reference models renders the precise and dynamic profiling of DNA methylation patterns at these loci much less feasible.

In conclusion, we discussed several mechanisms through which DNA methylation may contribute to the maintenance of chromosomal stability by acting at the centromere level. We believe many other possible, as of yet unidentified, ways exist. These likely involve both the direct mechanisms by which DNA methylation can shape chromatin structure, and indirect ones, based on the recruitment of accessory factors needed for proper centromere functions. Future studies are needed to reveal the exact mechanisms of action to keep centromeres under control.
